# Dendrimer-entrapped gold nanoparticles as potential CT contrast agents for blood pool imaging

**DOI:** 10.1186/1556-276X-7-190

**Published:** 2012-03-19

**Authors:** Han Wang, Linfeng Zheng, Rui Guo, Chen Peng, Mingwu Shen, Xiangyang Shi, Guixiang Zhang

**Affiliations:** 1 Department of Radiology, First People's Hospital, School of Medicine, Shanghai Jiao Tong University, Shanghai 200080, People's Republic of China; 2 College of Chemistry, Chemical Engineering and Biotechnology, Donghua University, Shanghai 201620, People's Republic of China; 3 State Key Laboratory for Modification of Chemical Fibers and Polymer Materials, Donghua University, Shanghai 201620, People's Republic of China

## Abstract

The purpose of this study was to evaluate dendrimer-entrapped gold nanoparticles [Au DENPs] as a molecular imaging [MI] probe for computed tomography [CT]. Au DENPs were prepared by complexing AuCl_4_^- ^ions with amine-terminated generation 5 poly(amidoamine) [G5.NH_2_] dendrimers. Resulting particles were sized using transmission electron microscopy. Serial dilutions (0.001 to 0.1 M) of either Au DENPs or iohexol were scanned by CT *in vitro*. Based on these results, Au DENPs were injected into mice, either subcutaneously (10 μL, 0.007 to 0.02 M) or intravenously (300 μL, 0.2 M), after which the mice were imaged by micro-CT or a standard mammography unit. Au DENPs prepared using G5.NH_2 _dendrimers as templates are quite uniform and have a size range of 2 to 4 nm. At Au concentrations above 0.01 M, the CT value of Au DENPs was higher than that of iohexol. A 10-μL subcutaneous dose of Au DENPs with [Au] ≥ 0.009 M could be detected by micro-CT. The vascular system could be imaged 5 and 20 min after injection of Au DENPs into the tail vein, and the urinary system could be imaged after 60 min. At comparable time points, the vascular system could not be imaged using iohexol, and the urinary system was imaged only indistinctly. Findings from this study suggested that Au DENPs prepared using G5.NH_2 _dendrimers as templates have good X-ray attenuation and a substantial circulation time. As their abundant surface amine groups have the ability to bind to a range of biological molecules, Au DENPs have the potential to be a useful MI probe for CT.

## Introduction

Molecular imaging [MI] combines conventional imaging technologies with MI probes, which are designed to detect aspects of biochemistry and cell biology that underlie disease progression and treatment response [1-5]. MI includes optical imaging, nuclear-based imaging (both positron-emission tomography and single photon emission tomography), and magnetic resonance imaging. Due to the difficulty of designing suitable contrast agents and probes, the use of X-ray computed tomography [CT] in MI has been limited. However, CT affords better spatial and density resolutions than other imaging modalities. These advantages become particularly apparent when CT is used to diagnose diseases in the thorax, such as lung cancer. There thus exists an urgent need to enhance the capabilities of CT by developing suitable MI probes.

Gold nanoparticles [AuNPs] have seen increasing use recently in cancer imaging and treatment [6-18] as they offer several advantages over conventional, iodine-based X-ray or CT contrast agents. First, because of its higher atomic number and electron density, gold has a higher X-ray absorption coefficient than iodine, endowing it in principle with a greater ability to enhance contrast on CT [19]. AuNPs also appear to be biocompatible [20,21]. It is relatively easy to modify the surface of AuNPs with functional groups such as targeting molecules or specific biomarkers, endowing the resulting particles with characteristics favorable for a range of MI applications [22-24]. Finally, proper treatment of AuNPs can increase their circulation time in the cardiovascular system [CVS] by allowing them to avoid removal by the reticuloendothelial system [RES] [22,25,26]. This is particularly advantageous when treating tumors, whose combination of leaky vasculature and poor lymphatic drainage can result in what is known as the enhanced permeation and retention [EPR] effect. Extended circulation times can exploit the EPR effect to enhance transport of AuNPs to the tumor site, while in parallel, the particles' bound targeting molecules increase the rate of endocytotic uptake [22,25].

Dendrimers are a class of highly branched, synthetic, and spherical macromolecules comprising a wide array of types, chemical structures, and functional groups [27]. Two types are commercially available: the poly(amidoamine) [PAMAM] and poly(propylene imine) dendrimers. Both types can be synthesized to different generations, each generation increasing proportionally in size and molecular weight. Thus, for example, generation 4 [G4] PAMAM dendrimers are approximately twice the size of generation 3 [G3]. These compounds have several advantages for clinical use. Not only are they highly soluble in aqueous solutions, but also their size can be precisely controlled. In addition, the terminal amine groups can easily be acetylated to shield their positive potential, thereby avoiding nonspecific binding and toxicity [28,29]. Dendrimers also possess a hollow interior that can be used to trap AuNPs as well as a substantial number of available surface amino groups that can be modified by a range of targeting molecules [30-32]. Dendrimers thus have a considerable potential as a nanoplatform to create multifunctional, dendrimer-entrapped gold nanoparticles [Au DENPs] [33,34], which have the additional benefit of being stable not only in water, phosphate-buffered saline [PBS], and cell culture medium, but also at different temperatures and pH conditions [35]. In this study, we synthesized and characterized Au DENPs and performed a preliminary evaluation of their ability to attenuate X-rays *in vitro *and their *in vivo *use as a MI probe for CT.

## Materials and methods

### Synthesis and characterization of Au DENPs

Generation 5 PAMAM [G5.NH_2_] dendrimers with a polydispersity index less than 1.08 were purchased from Dendritech (Midland, MI, USA). All other chemicals were obtained from Aldrich (St. Louis, MO, USA) and used as received. The water used in all the experiments was purified using a Milli-Q Plus 185 water purification system (Millipore, Bedford, MA, USA) with a resistivity higher than 18 MΩ cm. Regenerated cellulose dialysis membranes (molecular weight cutoff, 10, 000) were acquired from Fisher (Waltham, MA, USA).

Au DENPs were synthesized using previously reported methods [30,35,36] with minor variations. Briefly, the particles were prepared using sodium borohydride reduction chemistry, with gold salt/dendrimer molar ratios of 51.2:1 and 200:1. The formed Au DENPs were denoted as {(Au^0^)_51.2_-G5.NH_2_} DENPs and {(Au^0^)_200_-G5.NH_2_} DENPs. For intravenous injection, {(Au^0^)_51.2_-G5.NH_2_} DENPs were further acetylated to neutralize the surface charge of the particles to form {(Au^0^)_51.2_-G5.NHAc} DENPs. The characterization of {(Au^0^)_51.2_-G5.NH_2_} and {(Au^0^)_51.2_-G5.NHAc} DENPs has been reported elsewhere [36]. To determine the size distribution of the {(Au^0^)_200_-G5.NH_2_} DENPs, a 1 mg/mL aqueous solution of each sample was dropped onto a carbon-coated copper grid and allowed to air-dry. The grids were then viewed by transmission electron microscopy [TEM] using a JEOL 2010F analytical electron microscope (JEOL, Tokyo, Japan) operating at 200 kV. For each sample, 300 Au DENPs were randomly selected for size analysis, which was performed in parallel by three investigators. The size-distribution histogram was produced using ImageJ software (http://rsb.info.nih.gov/ij/download.html).

### *In vitro *CT imaging and CT value measurement

Fifteen serial dilutions of either {(Au^0^)_200_-G5.NH_2_} DENPs or iohexol (Omnipaque^®^, 300 mg iodine per mL, GE Healthcare, Milwaukee, WI, USA), ranging from 0.001 to 0.1 M of [Au] or [I], were prepared in 1.5-mL microcentrifuge tubes and placed in a self-designed scanning holder. The tubes were then scanned using a 64-row multidetector CT system (LightSpeed VCT, GE Medical Systems, Milwaukee, WI, USA) with the following parameters: tube voltage, 120 kV; tube current, 50 mA; slice thickness, 0.625 mm; slice space, 0; scan field of view, 25 cm; display field of view, 6 cm; matrix, 512 × 512. Each concentration of either {(Au^0^)_200_-G5.NH_2_} DENPs or iohexol was scanned three times, with a 24-h interval between any two scans.

Images were reconstructed, and CT values measured, using a GE imaging workstation (Advantage Workstation 4.3, GE Medical Systems, Milwaukee, WI, USA). Images were reconstructed in the axial plane, after which a 20-mm^2 ^circle was laid over the center of each image to define the region of interest for the measurement of CT value. CT values were calculated based on three scans of each sample, each performed by a different investigator, and the data were presented as mean ± standard deviation.

### *In vivo *CT imaging

The institutional animal care committee of Shanghai Jiao Tong University approved all animal experiments. BALB/c mice (20 to 25 g, Shanghai Laboratory Animal Center) were anesthetized by intraperitoneal injection of 3% sodium pentobarbital (35 mg/kg). The mice were scanned using a micro-CT imaging system (eXplore Locus, GE Healthcare, London, Ontario, Canada) set to the following parameters: tube voltage, 80 kV; tube current, 450 μA; exposure time, 400 ms; slice thickness, 45 μm; slice space, 0; scan field of view, 45 mm × 80 mm; effective pixel size, 0.046 mm. Images were reconstructed on a micro-CT imaging workstation (GEHC microView, GE Healthcare, London, Ontario, Canada) using the following parameters: voxel, 45 μm × 45 μm × 45 μm; display field of view, 10 to 25 mm.

To test the performance of Au DENPs as a CT MI agent, a 10-μL aliquot of {(Au^0^)_200_-G5.NH_2_} DENPs with [Au] of 0.007, 0.009, 0.01, or 0.02 M was subcutaneously injected into the back of the experimental mice while the control mice were injected with an equal volume of PBS (pH 7.4). These Au DENP concentrations were chosen on the basis of both the CT value of the soft tissue area in the uninjected mice and the previously derived CT value measurements of Au DENP solutions alone. After injection, micro-CT scans and image reconstruction were carried out as described above. Each experiment was carried out three times.

### *In vivo *dynamic digital X-ray photography

Dynamic digital X-ray photography was carried out using a mammography system (Senographe DS, GE Medical Systems, Milwaukee, WI, USA) set to the following parameters: tube voltage, 22 kV; tube current, 8 mA; exposure time, 400 ms. The anesthetized mice were imaged before injection and then 5, 20, and 60 min after injection of either Au DENPs or iohexol. The acetylated {(Au^0^)_51.2_-G5.NHAc} DENPs (300 μL, [Au] = 0.2 M), 300 mg/mL iohexol, or PBS were injected into the tail vein at a flow rate of 300 μL/min. Images were interpreted on a picture archiving and communication system monitor (Pathspeed, GE Medical Systems Integrated Imaging Solutions, Mt. Prospect, IL, USA) after adjustment of the optimal window settings, and then analyzed. This part of the study was performed by two investigators in consensus.

## Results

### Synthesis and characterization of Au DENPs

**Figure 1:**
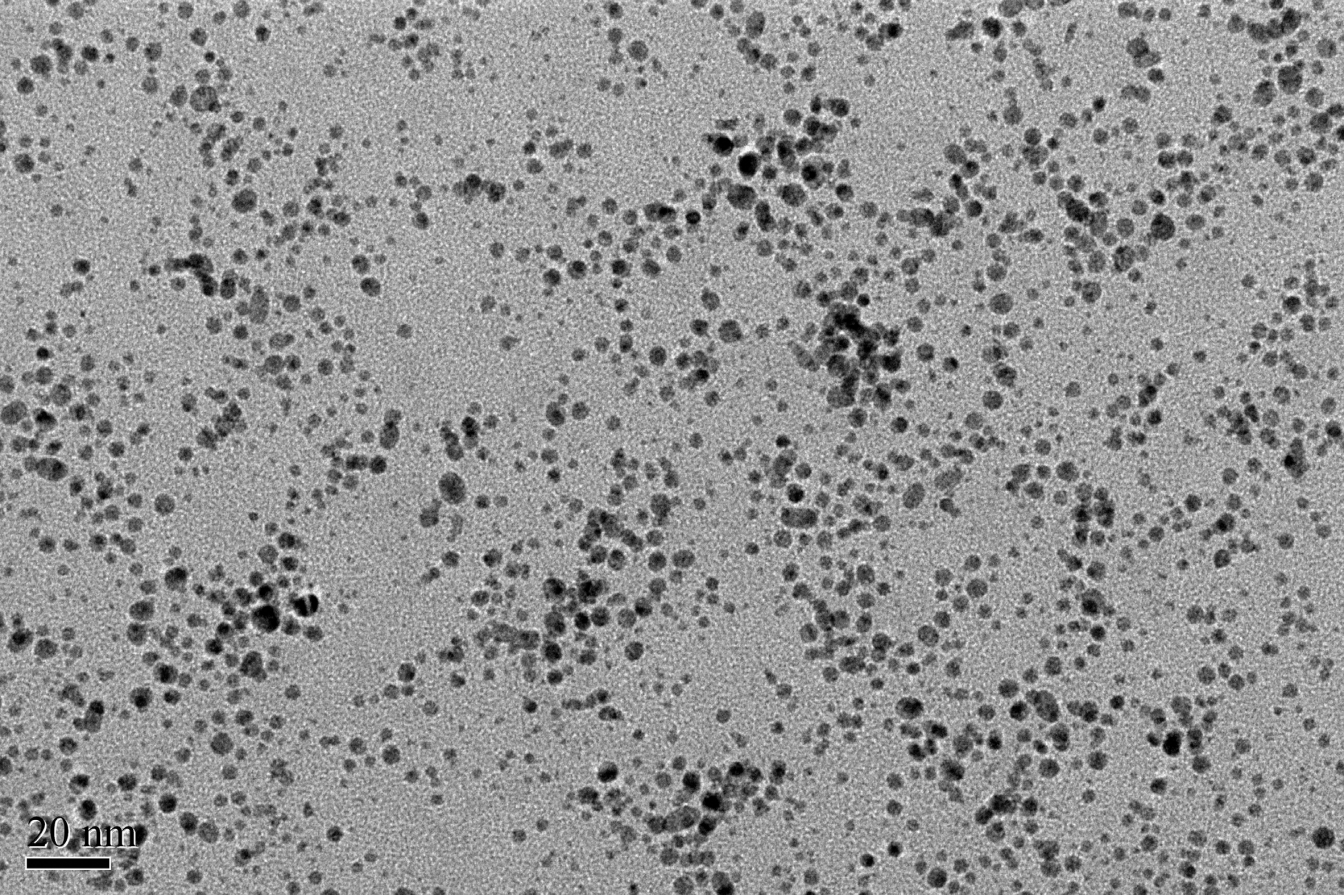
**TEM images of {(Au^0^)_200_-G5.NH_2_} DENPs**.

**Figure 2:**
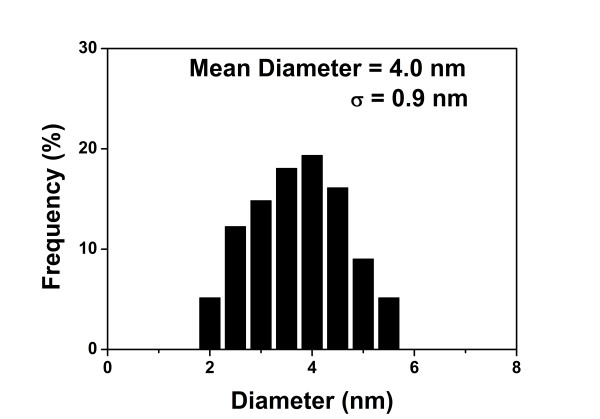
**Size-distribution histogram of {(Au^0^)_200_-G5.NH_2_} DENPs**.

Figure 1 shows a typical TEM image of the synthesized Au DENPs prepared with a gold salt/dendrimer molar ratio of 200:1. The size of the {(Au^0^)_200_-G5.NH_2_} DENPs was estimated to be 4.0 ± 0.9 nm. The size-distribution histogram (Figure 2) shows that the particles were relatively uniform in size, forming a normal distribution.

### *In vitro *CT imaging and CT value measurement

**Figure 3:**
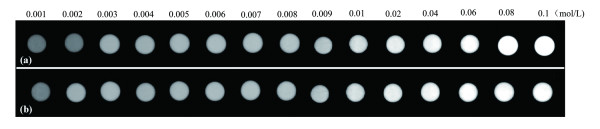
**Axial CT images**. {(Au^0^)_200_-G5.NH_2_} DENPs (a) and iohexol (b) at a range of concentrations in 1.5-mL microcentrifuge tubes.

**Figure 4:**
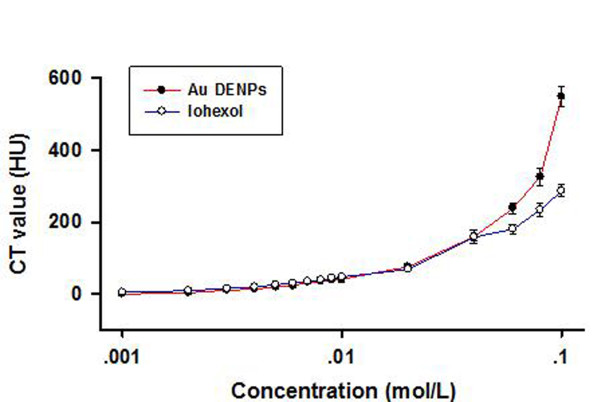
**Concentration-CT value curves of {(Au^0^)_200_-G5.NH_2_} DENPs and iohexol**.

The reconstructed CT images obtained by scanning various concentrations of either {(Au^0^)_200_-G5.NH_2_} DENP or iohexol solutions are shown in Figure 3. CT values (in Hounsfield units [HU]) derived from these scans (Table 1) were used to construct the concentration-CT value curves shown in Figure 4. These showed, first, that at a concentration of 0.01 M or less, X-ray attenuation by Au DENPs was slightly less than that observed with an iohexol solution containing the same concentration of iodine. However, these differences were small, within 6 HU. In contrast, as the concentration was increased above 0.01 M, X-ray attenuation by Au DENPs became progressively greater than that of the corresponding iohexol solution.

**Table 1:** CT values of Au DENPs and iohexol solutions.

Concentration (M)	CT value (HU)	
	Au DENPs	Iohexol
0.001	0.9 ± 1.9	5.1 ± 2.8
0.002	4.2 ± 2.3	10.3 ± 2.8
0.003	11.6 ± 2.1	16.3 ± 1.3
0.004	14.9 ± 1.4	20.8 ± 1.5
0.005	20.1 ± 2.2	26.8 ± 1.6
0.006	23.3 ± 1.4	31.2 ± 1.4
0.007	33.7 ± 1.5	35.6 ± 2.3
0.008	35.8 ± 1.9	40.1 ± 2.2
0.009	39.2 ± 2.9	44.9 ± 2.3
0.01	42.3 ± 8.7	48.3 ± 2.2
0.02	76.3 ± 5.8	69.1 ± 3.7
0.04	159.7 ± 18.7	158.6 ± 10.2
0.06	238.1 ± 15.6	180.7 ± 12.5
0.08	325.3 ± 23.3	233.9 ± 18.2
0.1	546.7 ± 27.1	286.5 ± 16.7

CT, computed tomography; Au DENPs, dendrimer-entrapped gold nanoparticles.

### *In vivo *CT imaging

**Figure 5:**
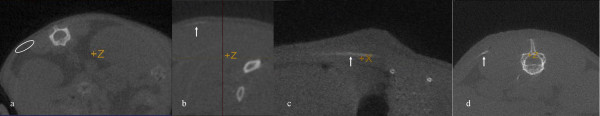
**Micro-CT images of the experimental mice**. The mice were injected subcutaneously with 10 μL {(Au^0^)_200_-G5.NH_2_} DENPs at [Au] of 0.007 (**a**), 0.009 (**b**), 0.01 (**c**), and 0.02 M (**d**). The white circle in (a) indicates the injection site. Arrows in the remaining panels show where Au DENPs have become distributed as a short segment in the interspace between the skin and the subcutaneous soft tissue. The mean CT values at the injection region were 31.57 (a), 41.23 (b), 48.56 (c), and 75.76 HU (d).

Figure 5 shows that {(Au^0^)_200_-G5.NH_2_} DENPs ≥ 0.009 M could be detected by micro-CT imaging after being injected subcutaneously into the *dorsum *of the mice [35]. After injection, the Au DENPs tended to become distributed as a short segment in the interspace between the skin and the subcutaneous soft tissue.

### Dynamic digital X-ray photography

**Figure 6:**
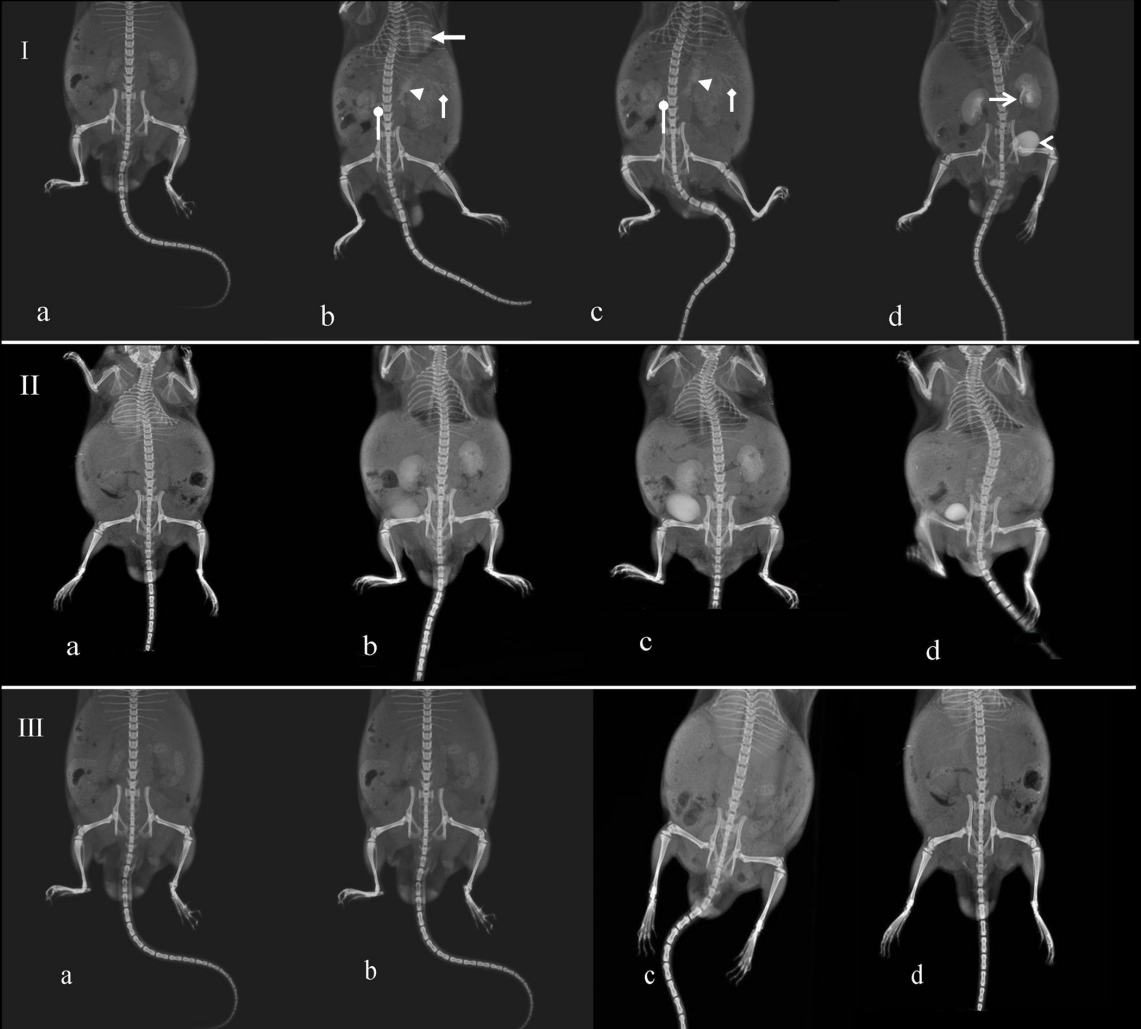
**Planar projection images after intravenous injection of {(Au^0^)_51.2_-G5.NHAc} DENPs or iohexol**. Rows I, II, and III contain images obtained after injection of the Au DENPs, iohexol, and PBS, respectively. In each row, image (**a**) is the pre-contrast image, (**b**) was taken 5 min after contrast injection, (**c**) at the 20-min, and (**d**) at 60 min after contrast injection. The following structures could be clearly distinguished 5 min after Au DENP injection (I-b): the heart (arrow), renal vein (oval arrow), main portal vein (arrow head), and branches of the portal vein (diamond arrow). At 20 min (I-c), the renal vein (oval arrow), main portal vein (arrow head), and branches of the portal vein (diamond arrow) remained distinct. Sixty minutes after Au DENP injection (I-d), the vascular system could no longer be visualized, but the urinary system, including the ureter (open arrow) and the urinary bladder (open arrow head), could be seen distinctly. After iohexol injection (II), the vascular system of the experimental mice could not be imaged. The urinary system began to be imaged 5 min after iohexol injection (II-b), and after 60 min (II-c), only the urinary bladder was defined.

At the 5- and 20-min time points after injection of {(Au^0^)_51.2_-G5.NHAc} DENPs, the vascular system of the mice was visible on CT (Figure 6I-b, 6I-c), with the heart, renal vein, main portal vein, and branches of the portal vein each clearly evident. The urinary system could be distinguished at the 60-min time point, with the ureter and urinary bladder defined clearly. In contrast, injected iohexol was unable to image the vascular system. At the 5- and 20-min time points after iohexol injection, the urinary system was imaged only vaguely, and after 60 min, only the urinary bladder was imaged. Together, these findings indicated that Au DENPs remained in the vascular system longer than iohexol and provided superior imaging enhancement.

## Discussion

In this study, we compared the ability of Au DENPs and iohexol to attenuate X-rays *in vivo *and *in vitro *as well as their ability to persist in the circulation after intravenous injection. We found a normal {(Au^0^)_200_-G5.NH_2_} DENP size distribution around 4.0 ± 0.9 nm. The CT value of {(Au^0^)_200_-G5.NH_2_} DENPs exceeded that of iohexol at Au concentrations above 0.01 M. {(Au^0^)_200_-G5.NH_2_} DENPs ([Au] ≥ 0.009 M, 10 μL) were detectable by micro-CT after subcutaneous injection. The vascular system could be imaged 5 and 20 min following the injection of {(Au^0^)_51.2_-G5.NHAc} DENPs into the tail vein, and the urinary system could be imaged after 60 min.

AuNPs hold a considerable promise as CT contrast agents for blood pool imaging because AuNPs persist longer in the circulation and exhibit a five- to seven fold higher attenuation of X-rays as compared with iodine-based agents [6,26,37]. According to the Lambert-Beer law [38], the relationship among an input X-ray flux *I*_0_, a tissue matrix of thickness *T *with linear attenuation coefficient *μ*_m_, and the transmitted flux *I*_m _is described by the formula *I*_m _= *I*_0_·e^-*μ*m*T*^. When both tissue and contrast agent are present, the flux *I*_c _that passes through a scanned section of thickness *t *is *I*_0_·e^-*μ*m*T*-*t*^·e^-*μ*c*t*^, or *I*_m_·e^-(*μ*c-*μ*m)*t*^, where *μ*_c _is the linear attenuation coefficient of the contrast agent. The difference in the signal between the surrounding matrix and the feature defined by the contrast agent can then be calculated as *C *= (*I*_m _- *I*_c_)/*I*_m _= 1 - e^-(*μ*c-*μ*m)*t*^. Thus, the difference in signal intensity induced by a contrast agent is introduced depending only on the thickness of the contrast agent and the difference in the linear attenuation coefficients of the contrast agent and the matrix. For this reason, the attenuation coefficient of a given contrast agent is one of the most important factors that determine its CT imaging efficiency.

Comparison of the concentration-versus-CT value curves of {(Au^0^)_200_-G5.NH_2_} DENPs and iohexol indicated that increasing the molar concentration of either element led to an increase in its attenuation coefficient. This was likely due to a concentration-dependent effect caused by the change in mass ratio between water molecules and either [Au] or [I]. The CT value of Au DENPs indicated that they had the superior ability to attenuate X-rays. Together, these results indicated that Au DENPs had a significant potential for use in CT MI based on their ability to enhance contrast. To further explore the feasibility of using Au DENPs in CT MI, we used {(Au^0^)_200_-G5.NH_2_} DENP solutions for micro-CT imaging and {(Au^0^)_51.2_-G5.NHAc} DENPs for dynamic digital X-ray photography *in vivo*. At concentrations above 0.009 M, Au DENPs had a much higher attenuation coefficient than the parenchyma, allowing very low-dose amounts of Au DENPs to be visible within the parenchyma on the CT image. We utilized acetylated {(Au^0^)_51.2_-G5.NHAc} DENPs for intravenous injection because acetylation of the terminal amines of Au DENPs can significantly improve their biocompatibility by avoiding the amine-induced toxicity that can arise at high concentrations [28,30,35,36]. Noting that although {(Au^0^)_200_-G5.NH_2_} DENPs are stable at PBS and cell culture medium [35], acetylation of {(Au^0^)_200_-G5.NH_2_} DENPs cannot generate stable {(Au^0^)_200_-G5.NHAc} DENPs. This is because the {(Au^0^)_200_-G5.NH_2_} DENPs have a relatively larger size (4.0 nm) when compared with {(Au^0^)_51.2_-G5.NH_2_} (2.1 nm) [36]. After acetylation to transfer dendrimer terminal amine groups to acetamide groups, the dendrimer tertiary amines cannot stabilize the larger Au DENPs entrapped within the dendrimers. The results obtained using both {(Au^0^)_200_-G5.NH_2_} and {(Au^0^)_51.2_-G5.NHAc} DENPs are comparable in terms of X-ray attenuation intensity since Au DENPs prepared using different gold salt/dendrimer molar ratios with a size range of 2 to 4 nm display similar X-ray attenuation at similar Au concentration [35].

Effective MI probes must have a sufficient half-life in the vasculature as enough of the agent must be transported to the target site to allow data collection [25,39]. This is especially true when MI agents are used for tumor diagnosis. Tumor vessel walls are incomplete and fragile, containing large gaps between the endothelial cells and the basement membranes [40]. This makes tumor neo-vessels highly permeable, allowing contrast agents to diffuse freely from the vasculature into the interstitial space. However, the half-life of a nanoparticle contrast agent is also determined by its size [27,41,42]. Molecular probes that are less than 3 nm in diameter, such as G1 or G2 dendrimers, leak easily across vessel walls into the surrounding tissue. Larger particles, between 3 and 5 nm in diameter, such as G3 or G4 dendrimers, are quickly excreted through the kidney, making them potentially useful as functional renal contrast agents. Agents between 5 and 8 nm diameter, such as G5 or G6 dendrimers, are retained in the circulation and are thus best suited for use as blood pool contrast agents or MI probes. However, when the diameter exceeds 20 nm, the agents are easily taken up by the RES within the liver and spleen.

In view of the previous considerations, and as the objective of this study was to produce a probe with a maximum half-life *in vivo*, we used G5.NH_2 _dendrimers as templates to prepare Au DENPs. The images obtained after injection of Au DENPs or iohexol indicate that Au DENPs remained in the circulation longer than iohexol.

Previous studies have shown that the entrapment of AuNPs within dendrimer templates does not influence the surface properties of the dendrimers [35]. Thus, the Au DENPs we synthesized would be expected to retain the native ability of the PAMAM dendrimer, allowing effective chemical modification with biologically active molecules [30].

Although the current results are promising, further experimental studies will be needed to ensure that these Au DENPs are effective and safe for clinical application. For example, the ability of these molecules to be modified so as to target particular organs and tissues should be evaluated, and the ability of organs and tissues to take up thus modified particles specifically measured. Before clinical application is considered, the potential toxicity must be ruled out.

This preliminary study demonstrates that Au DENPs prepared using G5.NH_2 _dendrimer templates have good X-ray attenuation and a substantial circulation time in the CVS. Their potential to be biologically and chemically modified [43], combined with ongoing improvements in computer technologies and the spatial resolution of CT scanners, is likely to make CT MI with these and related agents an increasingly important tool for diagnosis and drug delivery.

## Competing interests

The authors declare that they have no competing interests.

## Authors' contributions

HW carried out the design of the imaging studies and participated in the *in vitro *and *in vivo *imaging studies and the manuscript drafting. LZ participated in the imaging studies and the manuscript drafting. RG carried out the design of the nanoparticle studies and participated in the synthesis and characterization of Au DENPs and the manuscript drafting. CP participated in the synthesis and characterization of Au DENPs. MS participated in the design of the nanoparticle studies. XS and GZ conceived the study and participated in its design and coordination. All authors read and approved the final manuscript.
